# Coverage and determinants of HIV testing and counseling services among mothers attending antenatal care in sub-Saharan African countries: a multilevel analysis

**DOI:** 10.1186/s12889-024-18373-5

**Published:** 2024-03-27

**Authors:** Temam Beshir Raru, Bedasa Taye Merga, Alemayehu Deressa, Abdi Birhanu, Galana Mamo Ayana, Belay Negash, Mulugeta Gamachu, Addisu Alemu, Fila Ahmed Hassen, Ahmed Mohammed, Dawit Firdisa, Lemma Demissie Regassa

**Affiliations:** 1 School of Public Health, College of Health and Medical Sciences, Haramaya University, P.O. Box. 235, Harar, Ethiopia; 2 School of Medicine, College of Health and Medical Sciences, Haramaya University, Harar, Ethiopia; 3 Departments of Public Health, Rift Valley University, Harar, Ethiopia

**Keywords:** Coverage, Determinants, Counseling, HTC, HIV, Testing, ANC, Women, Sub-sahara Africa

## Abstract

**Background:**

HIV/AIDS is one of the top global public health threats that causes significant cases, deaths, and socioeconomic impact. Even though both HIV testing and counseling are identified as essential HIV interventions during pregnancy, large population-representative data shows that service coverage and determinants are limited. Therefore, this study aimed to assess the coverage and determinants of HIV testing and counseling services among pregnant mothers attending antenatal care services in sub-Saharan African countries using different nationwide data.

**Methods:**

This study was conducted on large national-representative data from the Demographic Health Survey (DHS) using multilevel analysis. Data extraction, cleaning, coding, and statistical analysis were performed using STATA version 17. Weighting was used to ensure the representativeness of the sample and to obtain reliable estimates and standard errors. The multivariable multilevel logistic regression model was used to identify the determinants of HIV testing and counseling during the antenatal care visit. Adjusted odds ratios with 95% confidence intervals were used to measure statistical significance.

**Results:**

A total of 83,584 women attending antenatal care were included in this study. HIV testing and counseling coverage in sub-Saharan Africa was found to be 62.87% with a 95% CI of 62.54–63.19%. The HIV testing and counseling determinants included being in the age group of 35–49 (AOR = 1.64; 95% CI: 1.46–1.83), secondary and above education levels (AOR = 1.50; 95% CI: 1.39–1.60), having at least four ANC visits (AOR = 1.85; 95% CI: 1.68–2.02), living in an urban area (AOR = 1.40; 95% CI: 1.30–1.52), and living in countries such as Rwanda (AOR = 6.19; 95% CI: 5.19–7.38) and Mauritania (AOR = 0.02; 95% CI: 0.01–0.03).

**Conclusion:**

This study revealed that HIV testing and counseling coverage was 62.87% in sub-Saharan Africa. Factors affecting the HIV testing and counseling coverage were age, education, frequency of antenatal care visits, residence area, and living in Rwanda and Mauritania. Therefore, to increase HIV testing and counseling coverage in sub-Saharan Africa, policymakers on maternal health and other stakeholders should work with an integrated approach with other sectors and give prior attention to modifiable factors such as promoting women’s education and the comprehensiveness of antenatal care follow-up services during the follow-up.

## Introduction

HIV/AIDS is one of the top global public health threats that causes significant cases, deaths, and socioeconomic impact. Due to the fragile healthcare system, insufficient manpower, and inadequate laboratory facilities, the disease is imposing multidimensional negative effects on human life [1, 2].

Despite the efforts made to fight HIV/AIDS in sub-Saharan Africa (SSA), the pandemic is contributing a lot to negatively affecting the socio-economic aspects of the population because of the associated death [1, 3]. SSA accounted for 70% of the global HIV cases [4]. Of the 15% of women aged 15–24 living with HIV, 80% of them live in SSA. In the region, more than 70% of them are young women.

To address the negative impact of HIV/AIDS, providing the testing service is foundational for managing, caring for, and presenting the case to reduce further incidences. Even if the service is low, both testing and counseling those who are expected to be tested are crucial [5]. Thus, it is recommended that providing testing and counseling services for HIV should be the standard guideline at all levels where the services can boost the health and well-being of the individual [6]. After the implementation of the services, the world witnessed that HIV testing and counseling (HTC) are very essential in the global efforts to scale up universal access to HIV treatment, care, and prevention strategies [7]. Accordingly, Ethiopia, including SSA, is putting the routine HIV testing and counseling services into action by using an opt-out approach, which means “provider-initiated HIV testing,” to realize high coverage of the services for all, particularly for pregnant mothers attending antenatal care services [8].

Moreover, HIV testing and counseling are majorly identified as crucial interventions for preventing new HIV infections and encouraging timely antiretroviral treatment (ART) initiation [9]. To enhance Prevention of Mother-to-Child Transmission (PMTCT), routine HTC be performed during the service rendered to pregnant mothers [10].

Several studies revealed that factors such as fear of receiving the test and already knowing one’s serostatus, issues of privacy [11], perceived need to obtain a partner’s permission to be detected, and lack of knowledge about HIV prevention and treatment [9], assuming that the test is optional, not discussing HIV testing with a partner before the ANC visit [12], attitude towards testing, and marital status [13] were determinants of HIV testing and counseling among pregnant mothers attending ANC services. However, “large population-based study shows that service coverage and determinants are limited. Therefore, the purpose of this study is to assess the coverage and determinants of HIV testing and counseling services among pregnant mothers attending ANC services in SSA countries using different nationwide data.

## Methods and materials

### Study settings and data source

This study was done by using secondary data from the DHS program in sub-Saharan African (SSA) countries. The data were obtained from the official database of the DHS program at www.measuredhs.com after authorization was granted via online request by explaining the purpose of our study. DHS is a nationally representative household survey that collects data on a broad range of health indicators like mortality, morbidity, fertility, contraceptive utilization, and maternal and child health [14]. Data from the DHS was combined from 2016 to 2020 for seventeen countries in the SSA. During the designated period, the most recent DHS of country-specific datasets were extracted. The 17 SSA countries from which data were extracted are Angola, Benin, Burundi, Cameroon, Ethiopia, Gambia, Guinea, Liberia, Mali, Mauritania, Malawi, Nigeria, Rwanda, Sierra Leone, Uganda, South Africa, and Zambia. To collect data that is comparable across countries in the world, the DHS program adopts standardized methods that involve uniform questionnaires, manuals, and field procedures. In this study, we used the individual record (IR) file to extract the outcome and independent variables. The surveys employed stratified two-stage cluster sampling technique. Detailed survey techniques and sampling methods used to collect data have been documented elsewhere [15].

### Population and sample size

The source population for this study was all reproductive-age women (15–49) years old in SSA, whereas all reproductive-age women (15–49) years old in SSA who were in the selected countries were the study population. For this study, we excluded women who did not give birth in the five years before each country’s survey, and women who did not have any ANC visits during their last pregnancy within five years before each country’s survey. In addition, specific women who have no record for the outcome variable were also excluded.

Accordingly, a total weighted sample of 83,584 people who fulfilled the inclusion criteria was included in this study. The sample from each country was Angola (4,758), Benin (2,904), Burundi (6,935), Cameroon (4,184), Ethiopia (3,144), Gambia (4,152), Guinea (2,725), Liberia (2,621), Mali (3,641), Mauritania (4,565), Malawi (8,602), Nigeria (12,343), Rwanda (4,681), Sierra Leone (4,461), Uganda (7,232), South Africa (1,647), and Zambia (4,986).

### Measurements

The outcome variable of this study was components of the HTC during the ANC visit. The outcome variable is binary, and it is coded as 1 if the woman received all three components of HTC. i.e., the women (i) received pretest HIV counseling, (ii) took an HIV test and obtained the result, and (iii) received post-test HIV counseling were coded as 0 otherwise.

**Media exposure** was created from the respondents’ exposure to newspapers, magazines, radio, and television. Accordingly, if the respondents had used at least one of them, we would consider them to have media exposure.

### Data management and analysis

Data extraction, cleaning, coding, and statistical analysis were performed using STATA version 17 software. Before analysis, the data were weighted to ensure the representativeness of the sample and to obtain reliable estimates and standard errors. Cross-tabulations, tables, and graphs were used to describe the data.

Given the hierarchical structure of the DHS data, women in one cluster might be more similar to one another than women in another. This may lead to a violation of the assumptions of observational independence and uniform variance among clusters. Therefore, in order to provide an impartial estimate and a dependable standard error, an advanced statistical model must account for the cluster variability.

Furthermore, multilevel mixed effect logistic regression was fitted, accounting for the outcome variable’s dichotomous nature. Bi-variable multilevel analysis was used to identify variables that are candidates for multi-variable multilevel analysis and checked for significance at a *p*-value < 0.25. Finally, a multivariable, multilevel logistic regression model was used to identify the determinants of HTC during the ANC visit. Adjusted odds ratios with 95% confidence intervals were used to measure statistical significance.

After selecting the possible explanatory variables for multivariable logistic analysis, we have fitted four different plausible models. These were the null model (model with no explanatory variables) which were used to verify community variance and provide evidence to assess random effects at the community level, model 1 (which consists of only individual variables), model 2 (which includes community-level variables), and the final model (model 3 contains both individual and community-level variables). Finally, the model with smaller Akaike Information Criterion (AIC) and Bayesian Information Criterion (BIC) was considered a parsimonious model. For measures of variation (random effects), the intra-cluster correlation coefficient (ICC), the proportional change in variance (PCV), and the median odds ratio (MOR) were used.

## Results

### Basic characteristics of women’s attending Antenatal care

A total of 83,584 women were included in this study. Most of the women, 38,704 (46.31%), were between the ages of 25 and 34, and the majority, 58,582 (70.09%), were married. Two-thirds (66.30%) of the women had media exposure, and the majority (52.415, or 63.34%) of them had access to a health facility. In addition, the majority (52,789, or 63.16%) of the women included had 4 or more ANC visits. This study found high coverage of HTC among married women, among women who had secondary and above education, and among those who were residing in an urban area. There is also high coverage of HTC-rich women and those who have two children (Table 1).

**Table 1:** Basic characteristics of women in sub-Saharan African Countries from 2016 to 2020 and Bi-variable multilevel mixed-Effect logistic regression in COR (*n* = 83,584)

Variables	Weighted Frequency (%)	HTC during ANC		COR (95%CI)
		No(%)	Yes(%)	
**Women’s Age (Years)**				
15–19	7,214 (8.63%)	3,255 (45.12%)	3,959 (54.88%)	**Ref.**
20–25	20,421 (24.43%)	7,523 (36.84%)	12,898 (63.16%)	1.41 (1.31–1.51) **
25–34	38,704 (46.31%)	13,905 (35.93%)	24,799 (64.07%)	1.69 (1.58–1.81) **
35–49	17,245 (20.63%)	6, 351 (36.83%)	10, 894 (63.17%)	1.62 (1.50–1.74)**
**Marital Status**				
Single	6,764 (8.09%)	2,031 (30.03%)	4,733 (69.97%)	**Ref.**
Married	58,582 (70.09%)	23,680 (40.42%)	34,902 (59.58%)	1.03 (0.94–1.09)
Living with partner	13,171 (15.76)%	3,814 (28.96%)	9,357 (71.04%)	1.16 (0.99–1.27)
Widowed/Divorced	2,295 (2.75%)	806 (35.11%)	1,489 (64.89%)	1.00 (0.87–1.16)
Separated	2,772 (3.32%)	703 (25.36%)	2,069 (74.64%)	1.12 (0.99–1.27)
**Educational Status**				
No education	25,574 (30.60%)	13,776 (53.87%)	11,798 (46.13%)	**Ref.**
Primary	30,607 (36.62%)	9,841 (32.15%)	20,766 (67.85%)	1.49 (1.42–1.57)**
Secondary and Above	27,403 (32.79%)	7,418 (27.07%)	19, 985 (72.93%)	2.11 (2.00-2.23)**
**Occupation**				
No occupation	24,183 (28.93%)	10,441 (43.18%)	13,742 (56.82%)	**Ref.**
Had Occupation	59,401 (71.07%)	20,593 (34.67%)	38,808 (65.33%)	1.33 (1.27–1.69)**
**Partners Education**				
No education	23,202 (32.35%)	13,281 (57.24%)	9,921 (42.76%)	**Ref.**
Primary	21,471 (29.93%)	6,508 (30.31%)	14,963 (69.69%)	1.79 (1.69–1.90)**
Secondary and Above	27,061 (37.72%)	7,704 (28.47%)	19,357 (71.53%)	2.13 (2.02–2.59)**
**Partners occupation**				
No Occupation	24,165 (28.91%)	10,434 (43.18%)	13,731 (56.82%)	**Ref.**
Had Occupation	59,419 (71.09%)	20,601 (34.67%)	38, 818 (65.33%)	1.33 (1.27–1.39)**
**Media Exposure**				
No	28,171 (33.70%)	11,741 (41.68%)	16,430 (58.32%)	**Ref.**
Yes	55,413 (66.30%)	19,293 (34.82%)	36,120 (65.18%)	1.41 (1.35–1.47)**
**Access to Health Facility (** *n* ** = 82,746)**				
A big problem	30,331 (36.66%)	11,863 (39.11%)	18,467 (60.89%)	**Ref.**
Not a big problem	52,415 (63.34%)	19,059 (36.36%)	33,357 (63.64%)	1.15 (1.10–1.20)**
**Wealth Index**				
Poor	33,821 (40.46%)	14,204 (42.00%)	19,617 (58.00%)	**Ref.**
Medium	17,211 (20.59%)	6,603 (38.37%)	10,608 (61.63%)	1.24 (1.17–1.30)**
Rich	32,552 (38.95%)	10,227 (31.42%)	22,325 (68.58%)	1.61 (1.52–1.70)**
**Number of children in a family**				
No children	780 (0.93%)	361 (46.30%)	419 (53.70%)	**Ref.**
One child	20,035 (23.97%)	7,420 (37.04%)	12,615 (62.96%)	1.31 (1.10–1.58)**
Two children	17,375 (20.79%)	6,086 (35.03%)	11,289 (64.97%)	1.56 (1.30–1.88)**
3–4 Children	25,608 (30.64%)	9,160 (35.77%)	16,448 (64.23%)	1.64 (1.36–1.97)**
Five and above	19,785 (23.67%)	8,007 (40.47%)	11,778 (59.53%)	1.56 (1.30–1.87)**
**Number of ANC Visit**				
One visit	5,024 (6.01%)	3,045 (60.62%)	1,979 (39.38%)	**Ref.**
Two visit	7,096 (8.49%)	3,452 (48.66%)	3,643 (51.34%)	1.41 (1.27–1.56)**
three visit	18,675 (22.34%)	6,786 (36.34%)	11,889 (63.66%)	2.07 (1.90–2.26)**
4 and above visit	52,789 (63.16%)	17,750 (33.63%)	35,039 (66.37%)	2.51 (2.31–2.73)**
**Residence**				
Rural	53,614 (64.14%)	20,721 (38.65%)	32,893 (61.35%)	**Ref.**
Urban	29,970 (35.86%)	10,313 (34.41%)	19,657 (65.59%)	1.36 (1.25–1.49)**
**Country**				
Angola	4,758 (5.69%)	1,847 (38.83%)	2,911 (61.17%)	0.49 (0.43–0.57)**
Benin	2,904 (3.47%)	1,633 (56.23%)	1,271 (43.77)	0.31 (0.27–0.36)**
Burundi	6,935 (8.30%)	2,746 (39.60%)	4,188 (60.40%)	0.64 (0.56–0.73)**
Cameroon	4,184 (5.01%)	1,374 (32.85%)	2,810 (67.15)	1.09 (0.94–1.28)
Ethiopia	3,144 (3.76%)	1,949 (62.00%)	1,195 (38.00%)	0.24 (0.21–0.28)**
Gambia	4,152 (4.97%)	1,962 (47.25%)	2,190 (52.75%)	0.46 (0.39–0.54)**
Guinea	2,725 (3.26%)	2,094 (76.84%)	631 (23.16%)	0.09 (0.07–0.11)**
Liberia	2,621 (3.14%)	1,065 (40.65%)	1,556 (59.35)	0.69 (0.58–0.83)**
Mali	3,641 (4.36%)	2,872 (78.88%)	769 (21.12%)	0.08 (0.07–0.10)**
Mauritania	4,565 (5.46%)	4,303 (94.27%)	262 (5.73%)	0.02 (0.01–0.028)**
Malawi	8,602 (10.29%)	866 (10.07%)	7,736 (89.93%)	5.43 (4.72–6.23)**
Nigeria	12, 343 (14.77%)	4,166 (33.75%)	8,177 (66.25%)	**Ref.**
Rwanda	4,681 (5.34%)	478 (10.22%)	4,203 (89.78%)	5.11 (4.33–6.02)**
Sierra Leone	4,461 (5.34%)	1,457 (32.66%)	3,004 (67.34%)	1.12 (0.97–1.28)
Uganda	7,232 (8.65%)	1,401 (19.38%)	5,831 (80.62%)	2.32 (2.03–2.64)**
South Africa	1,647 (1.97%)	218 (13.25%)	1,429 (86.75%)	3.86 (3.13–4.75)**
Zambia	4,986 (5.97%)	600 (12.04%)	4,386 (87.96%)	4.41 (3.76–5.17)**

**, Significant at *p*-value < 0.001

### HIV testing and counseling (HTC) during ANC visit in sub-saharan African countries

**Fig. 1:**
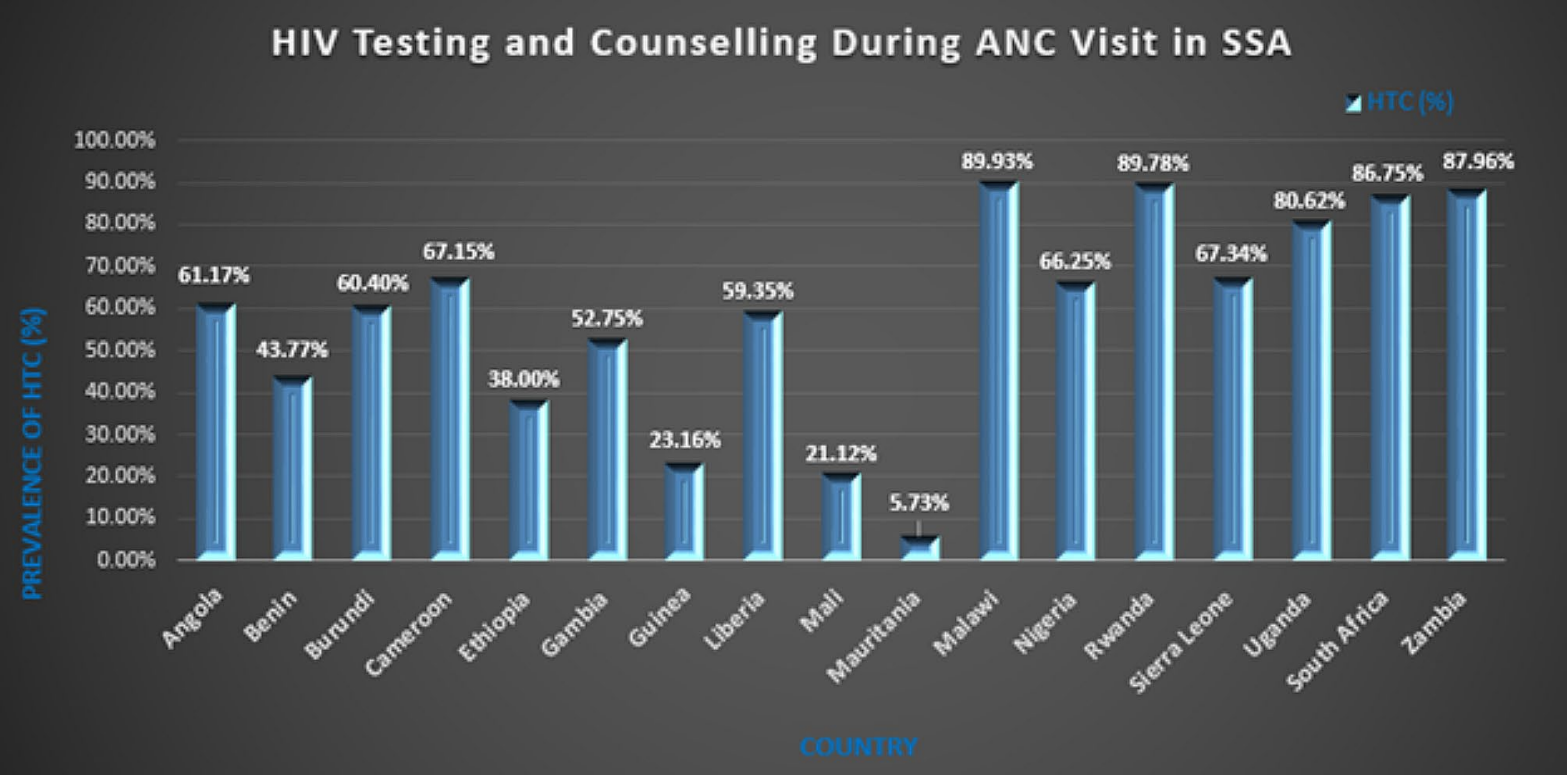
Prevalence of HIV Testing and Counseling (HTC) During ANC Visit in sub-Saharan Africa (SSA) Countries from 2016–2020

HTC coverage in sub-Saharan Africa was found to be 62.87% with a 95% CI (62.54–63.19). The highest and lowest HTC coverage were found in Malawi (89.93%) with a 95% CI of 89.28–90.55 and Mauritania (5.73% with a 95% CI of 5.09–6.44), respectively (Fig. 1).

### Determinants of HIV testing and counseling

#### Random effects

The results of the null model revealed that there was statistically significant variability in the odds of HTC with a community variance of 3.54, and the ICC in the null model suggested that only 5.83% of the total variability in HTC was ascribed to the differences between communities, which suggests there was variation in HTC among clusters. In the model-III model (model adjusted for both individual and community-level factors), community variance = 0.97; SE 0.03 remained significant but reduced, and 22.80% of the total variance of HTC can be attributed to the community, which suggests there were cluster differences. The value of the median odds ratio in the final model was 2.55, indicating the existence of cluster variation among individuals. It implies that a woman from a higher cluster was 2.55 times more likely to have HTC than a woman from a lower cluster. The PCV value for the better-fitted model was 72.60%, which shows that 72.60% of the variation in HTC was explained by combining variables in the final model (Table 2).

**Table 2:** Multivariable multilevel mixed-Effect logistic regression analysis of determinants of HIV testing and counseling in sub-Saharan Africa Countries from 2016 to 2020

Variables	Models			
	Null Model	Model-1 AOR (95% CI)	Model-2 AOR (95% CI)	Model-3 AOR (95% CI)
**Women’s Age (Years)**				
15–19	**-**	**Ref.**		
20–24	**-**	1.36 (1.23–1.48) **	**-**	1.32 (1.21–1.45) **
25–34	**-**	1.60 (1.45–1.76) **	**-**	1.58 (1.43–1.75) **
35–49	**-**	1.69 (1.51–1.89) **	**-**	1.64 (1.46–1.83) **
**Educational Status**				
No education	**-**	**Ref.**		
Primary	**-**	1.42 (1.34–1.50)**	**-**	1.17 (1.11–1.24)**
Secondary and Above	**-**	1.77 (1.66–1.89) **	**-**	1.50 (1.39–1.60)**
**Occupation**				
No occupation	**-**	**Ref.**		
Had Occupation	**-**	1.52 (0.39–5.90)	**-**	1.44 (0.42–4.92)
**Partners Education**				
No education	**-**	**Ref.**		
Primary	**-**	1.62 (1.53–1.72) **	**-**	1.13 (1.07–1.20)**
Secondary and Above	**-**	1.65 (1.55–1.75) **	**-**	1.23 (1.15–1.30)**
**Partners occupation**				
No occupation	**-**	**Ref.**		
Had Occupation	**-**	0.89 (0.23–3.48)	**-**	0.72 (0.21–2.47)
**Media Exposure**				
No	**-**	**Ref.**		
Yes	**-**	1.15 (1.10–1.21) **	**-**	1.25 (1.19–1.31)**
**Distance from health facility**				
A big problem	**-**	**Ref.**		
Not a big problem	**-**	1.07 (1.02–1.12) **	**-**	1.07 (1.02–1.11)**
**Wealth Index**				
Poor	**-**	**Ref.**		
Medium	**-**	1.09 (1.03–1.15) **	**-**	1.18 (1.11–1.24)**
Rich	**-**	1.20 (1.13–1.28) **	**-**	1.36 (1.27–1.45)**
**Number of children in a family**				
No children	**-**	**Ref.**		
One child	**-**	1.27 (1.01–1.60) *	**-**	1.21 (0.97–1.53)
Two children	**-**	1.41 (1.11–1.78) **	**-**	1.36 (1.08–1.71)**
3–4 Children	**-**	1.44 (1.13–1.82) **	**-**	1.38 (1.10–1.75)**
Five and above	**-**	1.43 (1.13–1.82) **	**-**	1.40 (1.12–1.76)**
**Number of ANC Visit**				
One visit	**-**	**Ref.**		
Two visit	**-**	1.46 (1.31–1.63) **	**-**	1.23 (1.10–1.37)**
three visit	**-**	2.04 (1.85–2.25) **	**-**	1.54 (1.39–1.70)**
4 and above visit	**-**	2.36 (2.15–2.58) **	**-**	1.85 (1.68–2.02)**
**Residence**				
Rural	**-**	**Ref.**		
Urban	**-**	**-**	2.02 (1.89–2.16)**	1.40 (1.30–1.52)**
**Country**				
Angola	**-**	**-**	0.42(0.37–0.48)**	0.51 (0.44–0.60)**
Benin	**-**	**-**	0.31(0.27–0.35)**	0.39 (0.34–0.45)**
Burundi	**-**	**-**	0.79(0.70–0.90)**	0.91 (0.80–1.04)
Cameroon	**-**	**-**	1.02(0.88–1.19)	1.10 (0.92–1.26)
Ethiopia	**-**	**-**	0.28(0.24–0.33)**	0.36 (0.31–0.42)**
Gambia	**-**	**-**	0.42(0.35–0.49)**	0.49 (0.41–0.58)**
Guinea	**-**	**-**	0.09(0.08–0.11)**	0.13 (0.11–0.15)**
Liberia	**-**	**-**	0.71(0.60–0.84)**	0.83 (0.69–1.01)
Mali	**-**	**-**	0.09(0.08-11)**	0.11 (0.09–0.14)**
Mauritania	**-**	**-**	0.02(0.01–0.03)**	0.02 (0.01–0.03)**
Malawi	**-**	**-**	6.65(5.79–7.63)**	7.83 (6.76–9.07)**
Nigeria	**-**	**Ref.**		
Rwanda	**-**	**-**	6.03(5.12–7.09)**	6.19 (5.19–7.38)**
Sierra Leone	**-**	**-**	1.17(1.02–1.34)**	1.49 (1.29–1.72)**
Uganda	**-**	**-**	2.70(2.37–3.08)**	2.86 (2.49–3.28)**
South Africa	**-**	**-**	3.41(2.78–4.19)**	3.72 (2.42–5.73)**
Zambia	**-**	**-**	4.76(4.07–5.57)**	5.35 (4.50–6.34)**
**Random Effects**				
Community Variance	3.54(0.09)	2.64(0.08)	1.03(0.03)	0.97(0.03)
ICC%	51.83%	44.51%	23.91%	22.80%
PCV	**Ref.**	25.42%	70.90%	72.60%
MOR	5.97	4.68	2.62	2.55
**Model Comparison**				
AIC	90781.77	76257.77	82923.20	**69781.07**
BIC	90800.44	76459.55	83100.53	**70138.78**

**, Significant at *p*-value < 0.0001

#### Fixed effects

The model with smaller AIC and BIC was considered a parsimonious model, and the interpretation of the fixed effects was based on this model. Model-III was adjusted for both individual and community-level factors that have small AIC and BIC, compared to other models, and this model fits the data well. In the multivariable analysis, respondent’s age group, respondent’s education, partner’s or husband’s education, media exposure, access to a health facility, wealth index, number of children in a family, number of ANC visits, place of residence, and country of origin were significant determinants of HTC in SSA at a 5% level of significance.

The odds of having HTC during an ANC visit were 1.64 (AOR = 1.64; 95%CI: 1.46–1.83) times higher among the women in the age group of 35–49, compared to the women in the age group of 15–19. Women who have secondary and above education levels were 1.50 times more likely to have HTC during ANC visits compared to those with no formal education (AOR = 1.50; 95% CI: 1.39–1.60). Women who have 4 and above ANC visits were 85% more likely to have HTC during ANC visits than their counterparts (AOR = 1.85; 95% CI: 1.68–2.02). Women who live in urban areas were 1.41 times more likely to have HTC during ANC visits compared to those who live in rural areas (AOR = 1.40; 95% CI: 1.30–1.52). The odds of having HTC during an ANC visit were 6.19 (AOR = 6.19; 95%CI: 5.19–7.38) times higher among the women who live in Rwanda compared to the women who live in Nigeria. However, the odds of having HTC during an ANC visit were 98% less likely among women who live in Mauritania, compared to those who live in Nigeria (AOR = 0.02; 95%CI: 0.01–0.03) (Table 2).

## Discussion

This study assessed the coverage and determinants of HIV testing and counseling services among mothers attending ANC in sub-Saharan African countries. This study found that the coverage of HIV Testing and Counseling Services among mothers attending ANC was 62.87% (95%CI: 62.54–63.19), and women’s age, mother’s education level, partner’s educational level, media exposure, distance from a health facility, wealth index, number of children in a family, number of ANC visits, residence, and country in which the mothers live determine the HIV testing and counseling of mothers at ANC clinics.

According to a finding of this study, the coverage of HTC among mothers attending ANC in sub-Saharan African countries was 62.87%. This finding was lower than the World AIDS Day report of 85% [16] and WHO global guidance on coverage of HIV testing and counseling for pregnant women of ≥ 95% [17, 18]. This could be because in sub-Saharan Africa, the strategy for testing and counseling services may be poor and not given attention. Another challenge in HIV testing and counseling service coverage is the acceptability of HIV services, which many developing countries with high HIV prevalence face. For many pregnant women, even after being diagnosed with HIV, there is no guarantee that they will accept treatment and adhere to it. As a result, making HIV testing and counseling services accessible and focusing on increasing coverage is essential for the elimination of infant HIV transmission in sub-Saharan Africa [19].

In this study, the odds of utilizing HTC among the women attending ANC in the age group of 35–49 were 1.64 times more likely than those women in the age group of 15–19. A similar study reported a relatively higher uptake of HIV testing among women, which is encouraging for women. The age group 25–34 had the highest likelihood of having been tested, and those age groups closely follow and understand that PMTCT services are well functioning [20]. Antenatal caregivers should emphasize the age group of pregnant mothers to reduce barriers to testing and increase awareness about HCT during ANC visits.

According to a finding of this study, women who have secondary and above education levels were 1.5 times more likely to utilize HTC compared to those with no formal education. Similarly, the study pointed out that the decisions of pregnant women to take up HTC are mostly influenced by a lack of information and a lack of education [21, 22]. The utilization of HCT is lowest among those with no formal education, and individuals who have primary education are about three times more likely to take HCT compared to those with no formal education [23]. Shreds of evidence of an absence of counselors, poor counseling, and a lack of awareness and knowledge about HCT are the main reasons cited for not undergoing HIV testing during pregnancy [24]. Similar findings depicted that pregnant mothers who had no formal education and had a primary level of education were less likely to accept human immune virus testing than women who had a diploma and an above-average level of educational status [25]. Hence, efforts must be directed towards groups of pregnant women who are less likely to be formally educated, and focusing on counseling during ANC utilization could be enormously effective in increasing the utilization of HCT among women.

In this study, Women who have 4 and above ANC visits were 1.85 times more likely to have HTC than their counterparts. A similar finding indicated that Pregnant women having only two antenatal care visits were 76% less likely to utilize the service as compared to pregnant women having three or more antenatal care visits [26, 27]. This highlights that focusing on improvement of quality and coverage of health services has significant effects on HCT service utilization of Pregnant Mothers. That means mothers who got good counseling and service during ANC visits trust any service given at the ANC clinic and this may be enhanced their HCT service utilization.

This study pointed out that women who live in urban areas were 1.4 times more likely to have HTC compared to those who live in rural areas. Another finding reported that the acceptance of provider-initiated HIV testing was higher among rural residents than in urban areas [28]. During counseling sessions, antenatal care providers should focus on barriers to provider-initiated HIV testing, such as residence, because rural residents could fear stigmatization and may not want to disclose their test results to their husbands. Therefore, strategies for educating and counseling rural residents on HCT should be developed and implemented to improve the utilization and changing behaviors of pregnant mothers.

This study also revealed that the odds of HTC were 6.2 times higher among the women who live in Rwanda compared to the women who live in Nigeria. However, the odds of HTC were 98% less likely among women who live in Mauritania compared to those who live in Nigeria. In Rwanda, HIV testing and counseling (HTC) is acceptable, and currently, using a rapid testing strategy is feasible and acceptable [29]. In Ethiopia, efforts should be made to improve the quality and coverage of HCT services and mitigate the barriers preventing mothers from seeking HIV testing [24]. African countries could be strengthening the introduction of HCT, which will lead to the diagnosis of HIV-positive women in labor, appropriate interventions, and the prevention of MTCT of HIV.

## Conclusion

This study revealed that HTC coverage was 62.87% in sub-Saharan Africa. Factors affecting the HTC coverage were age, education, frequency of ANC visits, residence area, and living in Rwanda and Mauritania. Therefore, to increase HTC coverage in SSA, policymakers on maternal health and other stakeholders should work with an integrated approach with other sectors and give prior attention to modifiable factors such as promoting women’s education and the comprehensiveness of ANC follow-up services during the follow-up.

## Data Availability

The EDHS datasets are publicly available up on request and can be accessed from the Measure DHS website (www.measuredhs.com) through an online request by explaining the objective of the study.

## Abbreviations

AIC: Akaike Information Criteria
ANC: Antenatal Care
AOR: adjusted odds ratio
ART: antiretroviral treatment
BIC: Bayesian Information Criteria
CI: Confidence Interval
COR: Crude Odds Ratio
DHS: Demographic and Health Survey
HTC: HIV Testing and Counseling
ICC: intra-cluster correlation coefficient
MOR: Median Odds Ratio
PCV: Proportional Change in Variance
SSA: sub-Saharan Africa
